# Case Fatality Rates Based on Population Estimates of Influenza-Like Illness Due to Novel H1N1 Influenza: New York City, May–June 2009

**DOI:** 10.1371/journal.pone.0011677

**Published:** 2010-07-21

**Authors:** James L. Hadler, Kevin Konty, Katharine H. McVeigh, Anne Fine, Donna Eisenhower, Bonnie Kerker, Lorna Thorpe

**Affiliations:** 1 Department of Health and Mental Hygiene, City of New York, New York, New York, United States of America; 2 School of Public Health, City University of New York, New York, New York, United States of America; INSERM, France

## Abstract

**Background:**

The public health response to pandemic influenza is contingent on the pandemic strain's severity. In late April 2009, a potentially pandemic novel H1N1 influenza strain (nH1N1) was recognized. New York City (NYC) experienced an intensive initial outbreak that peaked in late May, providing the need and opportunity to rapidly quantify the severity of nH1N1.

**Methods and Findings:**

Telephone surveys using rapid polling methods of approximately 1,000 households each were conducted May 20–27 and June 15–19, 2009. Respondents were asked about the occurrence of influenza-like illness (ILI, fever with either cough or sore throat) for each household member from May 1–27 (survey 1) or the preceding 30 days (survey 2). For the overlap period, prevalence data were combined by weighting the survey-specific contribution based on a Serfling model using data from the NYC syndromic surveillance system. Total and age-specific prevalence of ILI attributed to nH1N1 were estimated using two approaches to adjust for background ILI: discounting by ILI prevalence in less affected NYC boroughs and by ILI measured in syndromic surveillance data from 2004–2008. Deaths, hospitalizations and intensive care unit (ICU) admissions were determined from enhanced surveillance including nH1N1-specific testing. Combined ILI prevalence for the 50-day period was 15.8% (95% CI:13.2%–19.0%). The two methods of adjustment yielded point estimates of nH1N1-associated ILI of 7.8% and 12.2%. Overall case-fatality (CFR) estimates ranged from 0.054–0.086 per 1000 persons with nH1N1-associated ILI and were highest for persons ≥65 years (0.094–0.147 per 1000) and lowest for those 0–17 (0.008–0.012). Hospitalization rates ranged from 0.84–1.34 and ICU admission rates from 0.21–0.34 per 1000, with little variation in either by age-group.

**Conclusions:**

ILI prevalence can be quickly estimated using rapid telephone surveys, using syndromic surveillance data to determine expected “background” ILI proportion. Risk of severe illness due to nH1N1 was similar to seasonal influenza, enabling NYC to emphasize preventing severe morbidity rather than employing aggressive community mitigation measures.

## Introduction

The public health response to an emerging influenza pandemic, particularly whether to initiate aggressive community mitigation strategies such as school closure, depends in part on the severity of illness caused by the potentially pandemic strain: whether it has a more severe disease rate or higher mortality than usually seen with seasonal influenza [1]. Determining severity of illness as soon as possible after a potentially pandemic influenza strain is recognized is thus one of the highest priorities for public health authorities.

In the United States, a key measure for categorizing the potential severity of a pandemic strain is the case-fatality rate (CFR) among those infected [1]. The major challenge to directly calculating the CFR rate is specifying the denominator of how many people have been infected, given that most disease does not require hospital care, many persons do not and are advised not to seek medical attention, and strain-specific diagnostic testing capacity is likely to be limited.

Population-based telephone surveys can be a useful way to quickly assess and monitor the prevalence and distribution of influenza-like illness (ILI) in the community. However, they do not distinguish between influenza and other causes of ILI (e.g., respiratory syncytial virus, rhinoviruses, coronaviruses, parainfluenza viruses) and their use to rapidly estimate the prevalence of influenza is challenging.

On April 24, 2009, New York City (NYC) became the third geographic area of the US in one week to document the presence of novel H1N1 (nH1N1), later declared pandemic influenza (H1N1) 2009 [2], [3]. The first confirmed NYC cases occurred in students attending a high school in Queens, where approximately a third of the 2,700 students developed ILI during the course of a week [4]. In response, the NYC Department of Health and Mental Hygiene (DOHMH) implemented a multifaceted surveillance approach to understand the severity of illness, and the scope, distribution, and impact of nH1N1 in NYC [3], [5]. In addition to establishing surveillance for more severe illness including deaths and hospitalizations due to nH1N1, we used rapid, population-based telephone surveys to estimate the total burden of ILI and nH1N1 in NYC and, thus, enable rapid estimation of case-fatality and hospitalization rates.

This paper presents our estimates of the prevalence of nH1N1 during the 50 days of peak circulation of nH1N1 in NYC, the methods used to derive them, and the resulting case-fatality, hospitalization and intensive care unit (ICU) admission rates. To our knowledge, NYC was the only area of the country to directly obtain population-level ILI or nH1N1 prevalence during the initial spring 2009 wave of the pandemic.

## Methods

Methods used included serial population surveys to estimate ILI prevalence for the time period May 1–June 19, 2009, use of two separate methods to determine and then discount estimates for background ILI in order to estimate nH1N1 prevalence, and calculation of case-fatality, intensive care unit (ICU) admission and hospitalization rates using data from surveillance for deaths, ICU admissions and hospitalizations during this time period for numerators.

### Ethics

The investigation of this novel strain of influenza in April/May, 2009, including the population-based telephone surveys, was deemed public health practice and not human subjects research by both the General Counsel and the Institutional Review Board Chair of the New York City Department of Health and Mental Hygiene, and therefore did not require Institutional Review Board review.

### Population Surveys

DOHMH employed rapid telephone polling methods typically used in public opinion research to assess ILI prevalence among both adults and children during two overlapping periods in May and June 2009. We conducted two polls of approximately 1,000 households each, between May 20 and May 27, and between June 15 and June 19, 2009. This size sample was adequate to generate a reliable citywide prevalence estimate yet still be conducted quickly. Household samples of 1000 would typically yield data on more than 2500 persons. The predicted 95% margin of error around an estimated prevalence of 50% in such a sample is less than +/− 2%, even after adjusting for non-response weighting. We used a random-digit dialing telephone sampling methodology to obtain data from a random sample of residential households in NYC. A nonrandom adult from each household was asked to provide information on all household members. Interviews lasted 5 minutes and were conducted in both English and Spanish. Sampled numbers were dialed between five and six times to contact and interview a household, or until the sampled number was determined to be non-working. For the first survey, the Council of American Survey Research Organizations (CASRO) response rate 3 (RR3) was 8.4% with a cooperation rate of 31.2% [6]. For the second survey, the RR3 was 8.4% with a cooperation rate of 27.9%.

For each survey, the analysis dataset contained a record for every enumerated household member. Household members were linked by a household ID. Data were weighted to population estimates from the 2007 American Community Survey (ACS) [7]. Respondents were weighted to the 2007 ACS head of household distribution by borough (NYC has five boroughs), age group (0–17 years, 18–64 years, 65+ years), gender, number of persons in the household and race/ethnicity. Other household members were weighted to the non-head of household population by borough, age-group, household composition, and respondent's race/ethnicity to generate population estimates of ILI by age group.

ILI was defined as having fever and either cough or sore throat during the specified time period. In the first survey, respondents were asked whether they or other household members had experienced fever and either cough or sore throat between May first and the date of the interview (May 20–27). Information on household members was recorded by age group. In the second survey, the same procedures were used, but the time frame was changed to the past 30 days (May 15–19 to June 15–19).

The surveys included two overlapping time periods with different estimates of ILI prevalence. To combine the surveys, an estimate was needed of the proportion of ILI cases reported in each survey that occurred in the 13 day overlapping time period, May 15–27. Ideally, this should reflect the underlying epidemic curve for ILI in NYC during this time. To approximate this, we used emergency department (ED) visit data from our syndromic surveillance system. This system includes daily information on 92% of all hospital ED visits in NYC. Chief complaints are used to classify visits into syndrome categories, including an ILI category that utilizes a definition similar to the survey question, but which can also include the word “flu” [8], [9].

**Figure 1:**
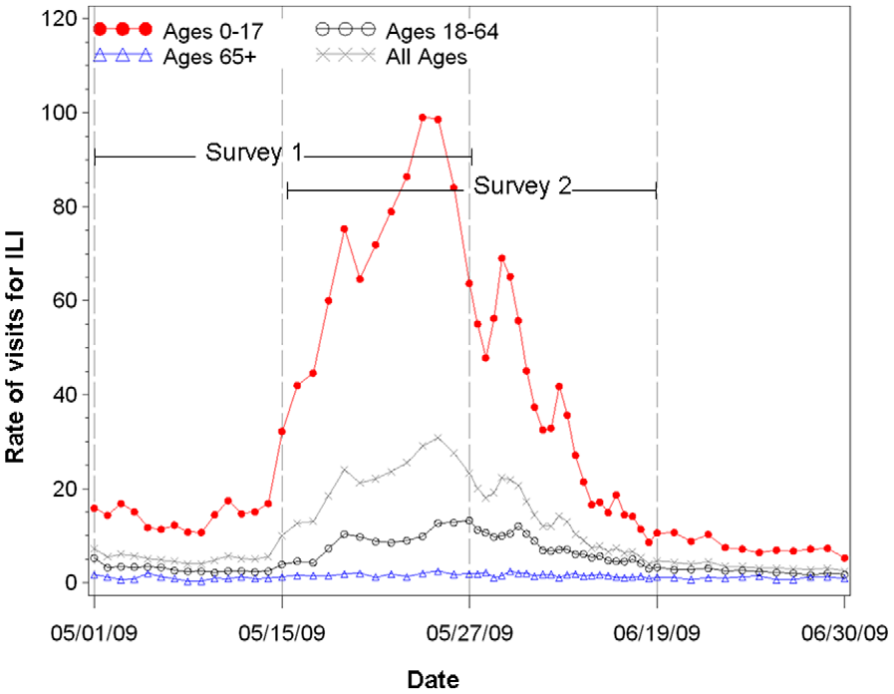
Overall and age-specific daily rates of ED visits for ILI, NYC, May–June 2009. Rate is number of emergency department (ED) visits for influenza-like illness (ILI) per 100,000 age-group specific population. Survey 1 was conducted from May 21–27 and measured ILI from May 1–27. Survey 2 was conducted June 15–19 and measured ILI from May 15 to June 19. The overlap period is from May 15–May 27.

For each survey, the number of ED ILI visits occurring during the non-overlap and overlap periods was calculated by age, sex, and borough. Assuming that the ILI prevalence reported in the survey followed the same distribution as the ED visits (Figure 1), the proportion of ILI from each survey that was in the overlap period could be estimated. The prevalence for the overlap period was then determined to be the value in the interval between two survey-specific estimates that best fit the ratios (overlap to non-overlap) implied by the ED ILI visits. The ILI prevalence estimates during each non-overlap survey period plus the overlap period were then combined.

### Adjustment for background ILI

Two approaches were used to estimate background ILI activity expected in the absence of nH1N1. Each approach produced overall and age group-specific estimates of background ILI activity. Each estimate of background ILI activity was applied to the combined survey data point prevalence to produce estimates of the percentage of NYC residents affected by nH1N1 from May 1 through June 19, 2009. Ninety-five percent confidence limits, adjusted for the complex survey design, were calculated around the adjusted point estimates.

#### Method 1: Using geographic differences in nH1N1 activity to estimate background ILI

During the reporting period of the May survey, results from ongoing enhanced surveillance of laboratory-confirmed hospitalized nH1N1 cases and of emergency department visits for ILI suggested that community transmission of nH1N1 was occurring primarily in certain parts of the city. Most cases were coming from the area in Queens surrounding the location of the initial high school outbreak, and from selected parts of Brooklyn. Assuming no geographic variability in the ratios of nH1N1 hospitalizations to nH1N1 prevalence and of nH1N1 prevalence to ILI prevalence, we hypothesized that ILI prevalence in the less affected areas would be largely attributable to causes other than nH1N1. Following this logic, we defined background ILI prevalence as the average prevalence from the initially less affected boroughs, Bronx, Manhattan and Staten Island from the first survey, overall and by age group, and assumed the same background rate for the entire survey period. The rates for the first survey period were calculated based on the distribution by date of responses to the May 20–27 survey, averaging approximately 23.1 days from May 1 and, thus, giving a 23.1 day prevalence. This estimate was then expanded to the full 50 day period. We then subtracted our estimates of background ILI from the combined ILI prevalence estimates to produce estimates of nH1N1 prevalence for the period May 1–June 19, 2009.

#### Method 2: Using auxiliary data from previous years to estimate background ILI

The second method used data from the ED syndromic surveillance system to adjust for background ILI. Using data from EDs reporting consistently over the preceding 5 years, we constructed an age group-specific Serfling model, similar to what is used for estimating excess mortality due to influenza. [10]. The Serfling models estimated the expected number of ILI visits in the absence of any type of influenza and were fit separately by age group and included seasonal terms, linear and quadratic trends, and day of week effects to adjust for differential health seeking behavior. This provided a baseline estimate of daily ILI visits in the absence of influenza for the period May 1^st^ to June 19^th^. Daily ED ILI visit data from 2009 were then used to reflect combined background ILI and nH1N1 activity and excess visits due to nH1N1 were calculated by subtracting the expected visits from observed values. The daily proportion of ED visits for ILI attributable to nH1N1 were then combined and applied to the ILI estimates for the three survey periods, the two non-overlapping periods and the overlap period, to estimate the prevalence of ILI due to nH1N1.

### Deaths, hospitalization and ICU admission surveillance

During this time period, active surveillance for the first 3 weeks followed by enhanced surveillance was conducted for deaths through all NYC hospitals and the medical examiner's office, including nH1N1 specific testing. For most of the period, enhanced surveillance for hospitalizations and ICU admissions due to nH1N1 was also conducted, including nH1N1 specific testing on persons hospitalized who tested influenza A positive and on all persons admitted to the ICU with acute respiratory illness, including ILI. Details of the surveillance efforts are described elsewhere [11], [12]. Eligible cases were persons who died or were hospitalized with laboratory-confirmed or probable (confirmed influenza A, not subtyped) nH1N1 infection with either onset of symptoms between May 1–June 19, 2009 inclusive or, in the absence of an onset date, first came to medical attention during this time period.

### Calculation of case-fatality, hospitalization and ICU admission rates

Two sets of case-fatality, hospitalization and ICU admission rates were calculated using numerators obtained through population-based nH1N1 death and hospitalization surveillance and each of the two sets of denominators of persons estimated to have had ILI due to nH1N1 from the population surveys after adjustment for background ILI. Overall and age group-specific rates were generated.

### Statistical Methods

ILI prevalence estimates and confidence intervals were produced using SAS-callable SUDAAN Statistical Methods software to adjust standard errors for complex survey design. Compound estimates such as the combined survey estimate of nH1N1 prevalence attributable ILI were generated using Monte Carlo methods with the R statistical package.

## Results

### ILI prevalence estimates

Table 1 presents the ILI prevalence estimates from each survey, as well as the combined estimates overall, by age-group and by borough.

**Table 1:** Overall and survey-specific ILI prevalence estimates by sex, age-group and borough, New York City, May 1–June 19, 2009.

	Survey 1*			Survey 2*			Combined Estimate May 1–June 19		
	Number with ILI**	Percent with ILI	95% CI	Number with ILI**	Percent with ILI	95% CI	Number with ILI**	Percent with ILI	95% CI
Total	576,000	6.9	6.0–7.9	1,007,000	12.0	10.0–14.6	1,318,000	15.8	13.2–19.0
Sex									
Male	266,00	6.7	5.3–8.5	402,000	10.1	7.5–13.3	540,000	13.5	10.2–18.0
Female	314,00	7.2	6.0–8.7	608,000	13.9	10.7–17.6	764,000	17.5	13.6–22.1
Age Group									
0–17	224,000	11.7	9.2–14.7	405,000	21.1	15.9–26.7	510,000	26.6	20.1–34.0
18–64	306,000	5.7	4.6–6.8	544,000	10.1	7.6–13.3	717,000	13.2	10.2–17.2
65+	45,000	4.3	3.0–6.3	59,000	5.7	4.1–7.1	91,000	8.8	6.2–12.6
Borough									
Bronx	50,000	3.6	2.2–5.7	127,000	9.1	5.8–13.4	155,000	11.1	7.0–16.6
Brooklyn	230,000	9.0	7.2–11.4	335,000	13.1	10.1–17.1	452,000	17.7	13.7–23.4
Manhattan	61,000	3.7	2.3–5.8	184,000	11.3	7.8–15.5	217,000	13.3	9.1–18.6
Queens	217,000	9.4	7.0–12.7	280,000	12.2	8.2–18.6	383,000	16.7	11.6–25.4
Staten Island	20,000	4.2	2.3–7.4	87,000	17.9	7.3–34.8	98,000	20.1	8.6–38.9

*Survey 1 conducted May 21–27, covering time period May 1–27. Survey 2 conducted June 15–19, covering time period May 15–June 19.

**Number with influenza-like illness (ILI) calculated by multiplying the group-specific 2007 population estimates by the percent with ILI and rounding to the nearest 1000.

Total ILI prevalence during the May 1–May 27 period was 6.9%. Prevalence was considerably higher in children (11.7%) than in adults (5.7%) or older adults (4.3%). There was geographic variability in ILI prevalence during this period. Prevalence was high in Brooklyn (9.0%) and Queens (9.4%) and lower in the three boroughs chosen to represent background ILI, Bronx (3.6%), Manhattan (3.7%) and Staten Island (4.2%).

Total ILI prevalence for the period May 15, 2009–June 19, 2009 was 12.0%, with age-related differences ranging from 21.1% in children 0–17 years to 5.7% in those 65 years and older. No variability in ILI prevalence by borough was observed during this time period.

After combining data from the two surveys, the estimated overall ILI prevalence was 15.8% (estimated N = 1,318,000 NYC residents), with age-specific estimates ranging from 26.6% among children ages 0–17 years, to 13.2% among adults 18–64 years and 8.8% among adults ≥65 years.

### Estimates of nH1N1 prevalence from combined ILI data

The results of each approach after adjusting for background ILI to obtain estimates of nH1N1 prevalence are presented in Table 2.

**Table 2:** nH1N1 prevalence estimates by adjustment method and age-group, New York City, May 1 to June 19, 2009.

	Combined Data May 1–June 19	Adjustment Method 1*			Adjustment Method 2**		
	Estimated ILI Percent Prevalence (95% CI)	Estimated Percent Background ILI (95% CI)	Estimated Percent nH1N1 Prevalence (95% CI)	Estimated Number with nH1N1 (95% CI)	Estimated Percent Background ILI (95% CI)	Estimated Percent nH1N1 Prevalence (95% CI)	Estimated Number with nH1N1 (95% CI)
NYC	15.8 (13.2–19.0)	8.0 (5.9–10.9)	7.8 (4.4–10.5)	639,000 (367,000–880,000)	3.6 (3.1–4.3)	12.2 (10.1–14.6)	1,017,000 (848,000–1,231,000)
Age-Group							
0–17 years	26.6 (20.1–34.0)	13.5 (8.4–21.5)	13.1 (4.5–20.2)	250,000 (87,000–388,000)	6.6 (5.0–8.5)	20.0 (15.1–25.5)	383,000 (290,000–488,000)
18–64 years	13.2 (10.2–17.2)	6.6 (4.4–9.9)	6.6 (2.6–9.4)	355,000 (156,000–548,000)	2.5 (2.0–3.2)	10.8 (8.3–14.0)	582,000 (446,000–758,000)
65+ years	8.8 (6.2–12.6)	5.6 (3.0–9.8)	3.2 (0.0–6.5)	34,000 (0–68,000)	3.7 (2.6–5.4)	5.1 (3.6–7.2)	53,000 (37,000–75,000)

*Adjustment Method 1 uses survey 1 data from the less affected boroughs to estimate background ILI.

**Adjustment Method 2 uses emergency department visit data for ILI from 2004–2008 to estimate background ILI.

The two methods gave nH1N1 point-prevalence estimates that were approximately 1.6-fold different. Method 1 resulted in an overall nH1N1 prevalence of 7.8%, with age-specific prevalence of 13.1% for children <18 years, 6.6% for adults 18–64 years, and 3.2% for those ≥65 years. By contrast, method 2 resulted in nH1N1 prevalence of 12.2% overall and 20.0%, 10.8%, and 5.1% in children, adults, and older adults, respectively.

### Case-fatality, hospitalization and ICU admission rates

Table 3 shows the overall and age-specific CFR, hospitalization and ICU admission rates for ILI due to nH1N1 by the two methods of adjustment for background ILI. The overall CFR ranged from 0.054 to 0.086 per 1,000 persons with ILI due to nH1N1. There was a strong association with age (p = 0.00001, chi-square for trend), with the CFR being more than 11-fold higher for those ≥65 years compared to children 0–17 years by each adjustment method. The CFR for those ≥65 ranged from 0.094 to 0.147 per 1,000 persons.

**Table 3:** Estimated case-fatality and case-hospitalization rates among persons with ILI due to nH1N1, by adjustment method, New York City, May 1–June19, 2009.

		Adjustment Method 1		Adjustment Method 2	
	No. Cases	No. persons nH1N1*	Rate/1000 persons nH1N1**	No. persons nH1N1*	Rate/1000 persons nH1N1**
Fatalities					
All	55	639,000	0.086	1,017,000	0.054
0–17 years	3	250,000	0.012	383,000	0.008
18–64 years	47	355,000	0.132	582,000	0.081
65+ years	5	34,000	0.147	53,000	0.094
Hospitalizations					
All	859	639,000	1.34	1,017,000	0.84
0–17 years	377	250,000	1.51	383,000	0.98
18–64 years	440	355,000	1.24	582,000	0.76
65+ years	42	34,000	1.24	53,000	0.79
ICU admissions					
All	214	639,000	0.335	1,017,000	0.210
0–17 years	81	250,000	0.324	383,000	0.211
18–64 years	122	355,000	0.344	582,000	0.210
65+ years	11	34,000	0.323	53,000	0.208

*Point estimate of number of persons with nH1N1 from Table 2.

**[No. cases]/[No. persons with nH1N1]×1,000.

The overall hospitalization rate ranged from 0.84 to 1.34 per 1,000 persons with ILI due to nH1N1. Children 0–17 years were at slightly higher risk for hospitalization than those who were older (RR = 1.22, 95% CI 1.06–1.39, p<0.01, adjustment method 1; RR = 1.30, 95% CI 1.13–1.48, p<0.001, adjustment method 2).

The overall ICU admission rate ranged from 0.21 to 0.34 per 1,000 persons with ILI due to nH1N1. There was no variation in ICU admission rates by age. However, the percentage of hospitalized cases who were admitted to the ICU did vary slightly by age. Adults 18 years and older were more likely than children 0–17 years to be admitted to the ICU (27.6% vs 21.5%, RR = 1.28, 95% CI 1.01–1.63).

## Discussion

It is critical to assess the severity of a potentially pandemic strain of influenza as soon as possible after it is recognized in order to inform the public and to guide public health response. Defining the risk of death following infection with a pandemic strain enables categorization of the potential severity of the pandemic, with a CFR of <1 death per 1000 persons infected being the criteria for the lowest severity, Category 1 pandemic strain [1]. Defining the risk of hospitalization enables projecting and planning for the burden on hospitals. To define these risks, it is essential to have both enough infections and a way to measure them, given that most influenza strains have a case-fatality rate of less than one per thousand symptomatic infections. In the first wave of pandemic influenza A H1N1 2009, New York City was in a position to attempt determination of CFR, given that it was particularly hard hit early. Using rapid polling techniques to obtain estimates of ILI, we had rough estimates of the number of nH1N1 infections within a week of completion of the first survey. At this time, surveillance for hospitalizations, observation of school outbreaks and the survey data collectively suggested that the outbreak was widespread, school-aged children were most affected, and the CFR appeared to be low. Thus, NYC adjusted response policies to focus on prevention and treatment of severe disease instead of community mitigation, including minimizing the number of school closings. Further, results of the first survey were released to the public to rapidly generate a more complete profile of illness burden and severity. After the second survey was completed, we shared survey findings without adjustment for background ILI with other researchers who were piecing together severity profiles from multiple cities to generate the multiplier model severity estimates that were initially published in September 2009 and which included preliminary NYC survey data (5). However, we needed sufficient time to measure deaths and hospitalizations and agreement on methods to combine the two surveys and adjust for background ILI before having confidence in the resulting specific estimates of case-fatality and hospitalization rates. Findings described here confirmed that the case-fatality rate was indeed low and well below 1 per thousand symptomatic infections for each age group, further supporting the policies adopted based on observations of school children.

Measuring influenza CFRs in “real-time” to inform public health efforts is a challenge that was acknowledged at the beginning of this pandemic [13], [14]. One method proposed in national pandemic planning materials was determination of the attack rate and CFR in a number of closed outbreaks and combining them [1]. However, this method was not used in this rapidly evolving pandemic. Instead, two different methods were used in the US. One used a multiplier model in which data from a number of sources were used to determine the ratio of medically attended visits to persons with ILI, the percentage of medical visits that were confirmed as due to pandemic influenza, the percentage of confirmed pandemic influenza infections that were hospitalized and the ratio of deaths to hospitalizations, with CFR and hospitalization rates then calculated using the respective multipliers [5], [15]. The other is the method described in detail in this paper but also applied to the crude initial ILI survey data from NYC in another paper [5], based on a direct estimation of the number of persons affected by pandemic influenza and the number of persons who died and/or were hospitalized who were confirmed to have pandemic influenza infection.

These methods have produced widely different measures of CFR, hospitalization and ICU admission rates due to pandemic H1N1 influenza in the US. The CFR ranges from a potentially low estimate of 0.05–0.09 per thousand based on our population survey data and 55 deaths, to a potentially high estimate of 0.48 to 5.1 per 1000 based on use of multipliers tied to 788 medically attended confirmed infections, 25 hospitalizations and 4 deaths in Milwaukee [5]. Age group-specific CFRs, hospitalization and ICU admission rates using these two methods also had approximately 10-fold higher estimates using the multiplier method.

Why were there such large differences between the two ways of estimating severity, especially since they have different implications for hospital preparedness? Is one method potentially more accurate than the other? We believe that the method used in NYC is more likely to produce an accurate measure of CFR simply because it is only dependent on two measures: population-level infection and deaths. The multiplier method is dependent on more measures and includes projection from the number of people diagnosed to the number seen for ILI and from the number seen to the number who were symptomatic. In this case, two additional factors could play a role: the numbers of confirmed cases and hospitalizations used in the multiplier model were small (788 and 25) and the data used to project from confirmed cases to estimate the population affected were obtained from studies done elsewhere and in special settings during the H1N1 pandemic (Chicago and Delaware) or from community surveys done when seasonal influenza was circulating [15].

We believe the NYC data, while still reflecting a range of estimates, to be fairly accurate for at least three reasons. First, numerator data were based on enhanced death surveillance including deaths referred to the medical examiner with specific testing for nH1N1 of all suspect deaths. Outpatient deaths were able to be identified and included. Second, despite low response rates on the survey, our ILI and nH1N1 prevalence data are consistent with data from other sources. The first survey showed a strong age-specific gradient with much higher prevalence in areas of NYC with initial amplification of nH1N1 and the data from the second survey were more uniform and much higher. These findings were consistent with hospitalization and ED syndromic surveillance data. In addition, the methods for adjustment for ILI likely produced artificially low and high estimates of nH1N1 prevalence, estimates that encompassed the actual prevalence. Assuming the baseline ILI prevalence in the absence of nH1N1 was the measured ILI in the first survey in boroughs with minimal nH1N1 activity based on hospitalization and ED syndromic surveillance data, we likely overestimated the background ILI rate and, correspondingly, had low nH1N1 prevalence estimates. Halfway through the first survey time period, it was clear from ED data that visits for ILI were increasing in those boroughs, a sign of nH1N1 activity spreading to them. Thus, the background ILI rates likely included nH1N1-related ILI. On the other hand, the ED syndromic surveillance adjustment method likely overestimated total ILI rates and, correspondingly, overestimated nH1N1 rates. In NYC, as in many other places in the US, people with ILI appeared to be more likely to go to the ED than normally would have in hopes of getting tested for nH1N1. Third, NYC clearly had a high nH1N1 prevalence during the first pandemic wave, one of the highest in the country based on death reports (25% of US reported nH1N1 deaths as of July 2, 2009) [16] and magnitude of ILI ED visits (peak of 16.5% of all ED visits on May 24 and 25 (unpublished data, NYC Department of Health and Mental Hygiene). The prevalence of nH1N1 infection was high enough to prevent a substantial second wave in the fall, during which activity in NYC was lower than in almost all other parts of the country (by week, peak of 4.4% of ED visits for ILI) [17]. If one projects from the NYC deaths occurring from May 1–June 19, 2009 using the median symptomatic CFR from the multiplier estimates (0.048% CFR) [5], then only 1.4% of NYC residents would have had ILI due to nH1N1 during our study period, a percentage which if doubled to 2.8% to account for asymptomatic infection would still be unlikely to produce much in the way of herd immunity. The estimates from the population survey which result in 7.8% to 12.2% of NYC residents having had symptomatic nH1N1 infection, especially if doubled to account for asymptomatic infection, are in a range that could explain the relatively low level of second wave activity.

Except for those 65 years and older, our estimates of age group-specific CFR for nH1N1 are almost identical to those derived from mortality data for seasonal influenza from 1990–1999, the data that forms the basis for the widely cited statistic that seasonal influenza causes an estimated 36,000 deaths per year in the US [18]. Assuming between 5% to 20% of the population is infected with seasonal influenza viruses each year, the annual CRFs per 1000 influenza infections from 1990–1999 are .01–.04 for 0–17 year olds, .03–.11 for 18–64 year olds, and 1.1–4.4 for those 65 and older. The range of age group-specific CFRs from nH1N1 in our study were .008–.012, .08–.13 and .09–.15, respectively. Given that nH1N1 attack rates were highest in younger persons who had the lowest CFR and that the number of deaths and CFR in the elderly were relatively low compared to seasonal influenza, the overall mortality from nH1N1 could be expected to be lower than that found in most influenza seasons. Our overall nH1N1 CFR ranged from .05–.09 per 1000 cases of ILI compared to a CFR for seasonal influenza ranging from 0.16–0.62 per 1000 influenza infections from 1990–1999, ranges encompassing 2.9 to nearly 7-fold differences. Given this mortality data, overall infection rates with nH1N1 would have to be 2.6–7-fold higher than with seasonal influenza to achieve average seasonal influenza mortality. The lack of increased severity of illness of a pandemic strain was not fully anticipated in most pandemic influenza planning scenarios, although it was accounted for in the influenza severity index developed by CDC in its Community Mitigation Strategy [1].

During the spring wave of nH1N1, it was noted in several journal articles that it is very difficult to measure case-fatality rates early in a pandemic [13], [14]. Based on our experience, we believe that it is possible to get such estimates during the peak of a first wave if not sooner using population-based survey methods to measure ILI prevalence in one or more geographic locations with clear evidence that thousands of infections are occurring (e.g., a substantial number of fatalities and clear increases in ILI). The challenges are to be able to adjust for background ILI rates and to time the survey(s) correctly to capture peak activity. In NYC, we were able to initially adjust for background ILI using rates of ILI in boroughs not heavily affected. However, in retrospect, we believe it is possible to use other measures of influenza activity, such as ED or sentinel provider visits to determine what percentage of ILI is due to causes other than influenza. The one time when such methods might be difficult to use is if a pandemic strain first emerged at a time when “background” ILI rates were particularly unstable from year to year, especially during the time periods when seasonal influenza usually occurs. An additional possible method to adjust for background ILI might have been to test a random sample of persons with ILI, including those who did not seek medical care, for nH1N1. However, that was logistically impossible at the time as all testing resources were focused on surveillance for severe illness and there was no serologic test available, Further, the percentage of ILI that was nH1N1 was likely constantly changing as the epidemic waxed and waned both overall and in different parts of the NYC. The overlapping time periods covered by the surveys made for an additional challenge to combine the data. Our desire to obtain contextual information early in the pandemic lead to rapid development and implementation of the first survey to measure ILI prevalence during the first three weeks of the pandemic. Realizing we might miss the peak without additional information, we initiated the second survey less than a month later and used a standard 30 day period to measure ILI prevalence. With 20-20 hindsight, a single well timed survey might have sufficed for measuring CFR but would not have provided the early data that indicated that nH1N1 had rapidly spread citywide and had affected 5–10% of the population in several boroughs. In the future, use of population-based surveys may be a faster and more accurate alternative than multiplier methods.

### Limitations

The major limitations of this study include the low survey response rate and the inability to measure nH1N1 prevalence directly. The low survey response rate was in part a predictable and necessary consequence of the rapidity with which the surveys were conducted, a limitation that can be expected whenever rapid polling methods are used. Additional survey-related limitations are those associated with any telephone survey, specifically recall, self-report and the potential for those with land line telephones to be different than those in the rest of the population. There are also limitations associated with conducting death surveillance and hospitalization surveillance. If a clinician does not think that a death could be influenza-related and/or fails to conduct testing or report to either public health authorities or the medical examiner, it will not be recognized and counted. Hospitalization surveillance relied in part on initial screening testing with an insensitive rapid antigen test for influenza A, and upon both clinician recognition of influenza and reporting in response to frequent telephone prompts. For ICU surveillance, however, only the clinical recognition of influenza and reporting was a limitation: nH1N1 specific testing was offered to all with acute respiratory illness, including ILI. These limitations all result in under measurement of fatalities and hospitalizations. The strengths of this approach include the rapid availability of the data, and the ability to capture data on children as well as adults. Of note, as of June 2010, no nH1N1 seroprevalence data on NYC residents following the Spring 2009 outbreak has become available to assess the accuracy of the prevalence estimates presented in this paper.
